# Association between muscle strength and advanced fibrosis in non‐alcoholic fatty liver disease: a Korean nationwide survey

**DOI:** 10.1002/jcsm.12598

**Published:** 2020-07-07

**Authors:** Sunyoung Kang, Min Kyong Moon, Won Kim, Bo Kyung Koo

**Affiliations:** ^1^ Department of Internal Medicine Seoul National University College of Medicine Seoul Korea; ^2^ Department of Internal Medicine Seoul National University Hospital Seoul Korea; ^3^ Division of Endocrinology, Department of Internal Medicine Seoul Metropolitan Government Seoul National University Boramae Medical Center Seoul Korea; ^4^ Division of Gastroenterology and Hepatology, Department of Internal Medicine Seoul Metropolitan Government Seoul National University Boramae Medical Center Seoul Korea

**Keywords:** Hepatic steatosis, Hepatic fibrosis, Muscle strength, Sarcopenia, Insulin resistance

## Abstract

**Background:**

We investigated the association between muscle strength and the prevalence of advanced fibrosis among individuals with non‐alcoholic fatty liver disease (NAFLD) using a nationwide cross‐sectional survey.

**Methods:**

Individuals, 20 to 79 years of age, from the Korean National Health and Nutrition Examination Surveys (KNHANES) from 2014 to 2016 were selected (*N* = 14 861), with sample weights applied. Muscle strength was quantified as the handgrip strength divided by the body mass index (BMI); low muscle strength (LMS) was defined as the lowest quartile (Q_1_) of the handgrip strength/BMI for our sample population. NAFLD was defined as hepatic steatosis index >36. Advanced fibrosis was defined as a fibrosis‐4 index score ≥1.30 (Fibrosis_FIB4_).

**Results:**

The mean age of the study population was 45.6 ± 0.2 years, and 42.4% were male. As muscle strength increased, the mean BMI and age decreased accordingly, and the proportions of diabetes, dyslipidaemia, hypertension, and obesity decreased significantly (*P* < 0.001 for all). In a crude analysis, the LMS was associated with an increased prevalence of NAFLD (odds ratio [OR] 3.62, 95% confidence interval [CI] 3.25–4.03, *P* < 0.001), which remained significant even after adjustment for age, sex, obesity, insulin resistance, diabetes, hypertension, dyslipidaemia, and high‐sensitivity C‐reactive protein (OR 1.66, 95% CI 1.28–2.16, *P <* 0.001). In this logistic regression model, the prevalence of NAFLD decreased by 24% with each quartile increment in muscle strength (OR 0.76, 95% CI 0.68–0.85, *P* < 0.001). Among individuals with NAFLD (*n* = 2092), LMS was significantly associated with the presence of advanced fibrosis (Fibrosis_FIB4_) independently of age, sex, obesity, diabetes, hypertension, dyslipidaemia, and high‐sensitivity C‐reactive protein (OR 1.66, 95% CI 1.01–2.49, *P* = 0.015), which lost its statistical significance after additional adjustment for insulin resistance.

**Conclusions:**

Low muscle strength is independently associated with NAFLD. The significant association between LMS and advanced fibrosis in NAFLD may be mediated through insulin resistance.

## Introduction

Sarcopenia is defined as a progressive decrease in muscle mass and function.^1^ Sarcopenia increases the risk for metabolic disease, physical disability, poor quality of life, and mortality.^2^, ^3^, ^4^ Recently, an independent association between appendicular skeletal muscle mass and histological severity of non‐alcoholic fatty liver disease (NAFLD) has been reported.^5^ Insulin resistance (IR), an important pathogenic mechanism of NAFLD,^6^ also increases the risk of sarcopenia via reduction of protein synthesis and increased protein breakdown.^7^, ^8^

To date, the association between NAFLD and sarcopenia has been principally evaluated from a perspective of low skeletal muscle mass rather than the quality of muscle function.^5^, ^9^ However, recent studies have demonstrated that muscle strength is more important than muscle mass in predicting health outcomes in older adults.^10^, ^11^ Yet there have been few population‐based studies on the association between muscle strength and NAFLD.

In the current study, we investigated the association between muscle strength and the prevalence of NAFLD through an analysis of a nationwide representative cross‐sectional survey dataset. In addition, the association between muscle strength and advanced fibrosis among individuals with NAFLD was also analysed.

## Materials and methods

### Study participants

We performed a retrospective analysis of the 2014 to 2016 data from the Korean National Health and Nutrition Examination Surveys (KNHANES). The KNHANES involves a nationwide cross‐sectional health examination and survey performed by the Korea Centers for Disease Control and Prevention in the Ministry of Health and Welfare, with details having been previously reported.^9^ Briefly, the KNHANES recruit participants using a stratified multistage probability‐based sampling design; sampling weights are assigned to each respondent to ensure that the results are representative of the whole Korean population.

**Figure 1:**
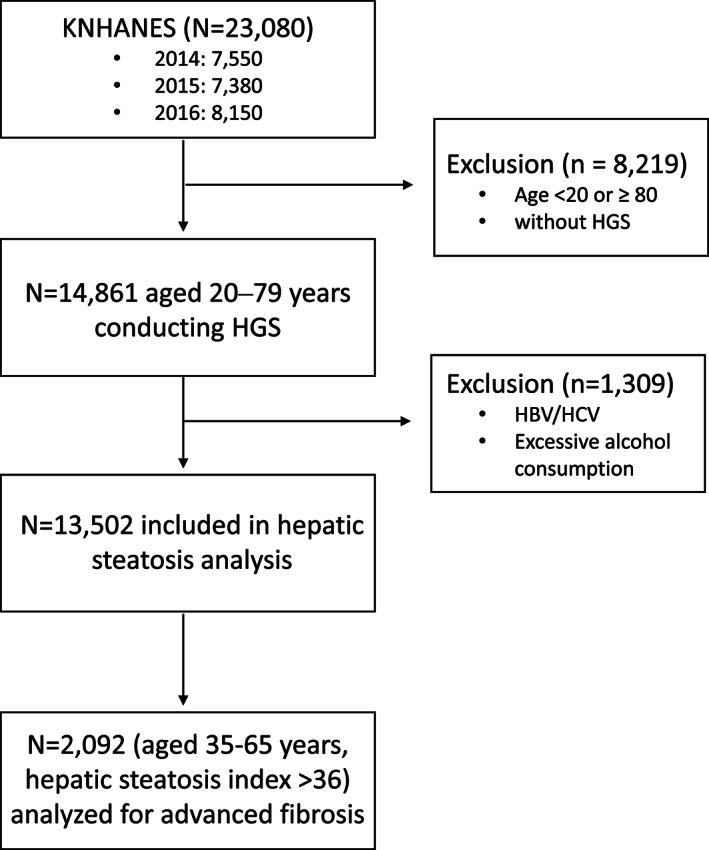
Flow diagram of participants for the study. Among a total of 23 080 participants in the Korean National Health and Nutrition Examination Surveys (KNHANES) 2014–2016, 13 502 individuals were included in our analysis. Individuals with non‐alcoholic fatty liver disease, defined by hepatic steatosis index, aged 35–65 years (*n* = 2092), were analysed for advanced fibrosis. HBV, hepatitis B virus; HCV, hepatitis C virus; HGS, handgrip strength.

Among a total of 23 080 participants enrolled in the KNHANES between 2014 and 2016, we included 14 861 individuals, 20 to 79 years old, whose handgrip strength (HGS) data were available. The exclusion criteria were as follows: alcohol consumption >210 g/week for men and >140 g/week for women,^12^ and positive serological markers for hepatitis B or C virus. After screening for the exclusion criteria, 13 502 individuals were included in our analysis (*Figure* 1). The use of the KNHANES 2014 data was approved by the institutional review board (IRB) of the Korea Centers for Disease Control and Prevention (IRB No. 2013‐12EXP‐03‐5C). IRB approval was not required for use of the KNHANES data between 2015 and 2016 under the Bioethics Act. Our study was performed in accordance with the ethical standards laid down in the 1964 Declaration of Helsinki and its later amendments.

### Metabolic parameters measurement

Diabetes mellitus (DM) was defined as an 8 h fasting plasma glucose level ≥7.0 mmol/L (126 mg/dL) or glycated haemoglobin level ≥48 mmol/mol (6.5%), or use of anti‐diabetic medication including insulin.^13^ Hypertension (HTN) was defined as a systolic blood pressure (BP) ≥ 140 mmHg, or diastolic BP ≥ 90 mmHg, or taking antihypertensive medications; dyslipidaemia (DL) was defined as non‐high‐density lipoprotein cholesterol ≥190 mg/dL or taking lipid‐lowering medication.^14^ Obesity and abdominal obesity were defined as a body mass index (BMI) ≥ 25 kg/m^2^ ^15^ and waist circumference ≥90 and ≥85 cm in men and women, respectively.^16^ Homeostatic Model Assessment of Insulin Resistance (HOMA‐IR) was calculated as [fasting glucose (mg/dL) × fasting insulin (uU/mL)]/405; IR was defined as HOMA‐IR ≥ 2.5.^17^ Elevated high‐sensitivity C‐reactive protein (hs‐CRP) was defined as ≥1 mg/L.^18^

### Assessment of muscle strength

Muscle strength was quantified by the HGS, assessed using a digital grip strength dynamometer (TKK 5401; Takei, Tokyo, Japan), as previously reported.^19^ Briefly, HGS was measured with the individual standing upright, with the elbow in full extension. Participants were instructed to squeeze the dynamometer as strongly as possible, for at least 3 s. Three measures were obtained, with a 1 min rest period between each trial. Following the previous studies, muscle strength was calculated from mean HGS of a dominant arm adjusted by BMI: HGS/BMI.^20^, ^21^ The quartiles of muscle strength were calculated in each gender, with Q_1_ and Q_4_ being the lowest and highest quartiles of HGS/BMI, respectively. The Q_1_ was defined as the group of subjects with low muscle strength (LMS).

### Assessment of non‐alcoholic fatty liver disease

Non‐alcoholic fatty liver disease was defined using previously well‐validated fatty liver prediction models: the hepatic steatosis index (HSI),^22^ calculated as 8 × alanine aminotransferase (ALT)/aspartate aminotransferase (AST) + BMI (+2, if diabetes; +2 if female). NAFLD was defined as HSI > 36.^22^ The sensitivity and specificity of HSI for prediction of NAFLD have been reported to be 86% and 66%, respectively, in Caucasians^23^ and 93.1% and 92.4%, respectively, in the Korean population.^22^

### Assessment of advanced fibrosis in non‐alcoholic fatty liver disease

Among individuals with NAFLD, as defined by the HSI, advanced fibrosis was assessed using the following prediction models: (i) fibrosis‐4 index (FIB‐4)^24^ = age (years) × AST [U/L]/(platelet [10^9^/L] × (ALT [U/L])^1/2^ and (ii) BARD score^25^ = the sum of the following scores (BMI ≥ 28 = 1 point; AST/ALT ratio ≥0.8 = 2 points; diabetes = 1 point). Advanced fibrosis was defined as either a FIB‐4 score ≥1.30 (Fibrosis_FIB4_)^26^ or a BARD score ≥2 (Fibrosis_BARD_).^25^ As the diagnostic performance of FIB‐4 for advanced fibrosis is unacceptably low in patients aged >65 or <35 years,^27^ only subjects aged 35–65 years (*n* = 2092) were included in analysis for advanced fibrosis.

### Statistical analyses

Variables are reported as the mean ± standard error (SE) or the prevalence ± SE (%). Linear regression or logistic linear regression analysis was used to compare the clinical variables according to muscle strength, adjusted for age, sex, obesity, IR, and other metabolic parameters. Sampling weights were applied to all analyses. Statistical analyses were performed using IBM SPSS Statistics (Version 22.0, IBM Corp., Armonk, NY, USA), with a *P* value <0.05 considered significant for all tests.

## Results

### Clinical characteristics according to muscle strength

The mean age of the study population was 45.6 ± 0.2 years, with 42.4% of the sample being male. Mean HGS was 23.7 kg (standard error [SE], 0.2 kg), 29.7 kg (SE, 0.2 kg), 33.4 kg (SE, 0.2 kg), and 37.5 kg (SE, 0.2 kg), and muscle strength (HGS/BMI) was 0.90 (SE, 0.0), 1.19 (SE, 0.0), 1.40 (SE, 0.0), and 1.71 (SE, 0.0) in the Q_1_, Q_2_, Q_3_, and Q_4_, respectively (*P* < 0.001; *Table* 1). As muscle strength increased, mean BMI and mean age were accordingly decreased (*P* < 0.001 in both; *Table* 1). Waist circumference, systolic BP, ALT, AST, triglycerides, glycated haemoglobin, hs‐CRP level, and HOMA‐IR also significantly decreased in order from the Q_1_ towards the Q_4_ (*P* < 0.001 in all; *Table* 1). As muscle strength increased, the proportions of cardio‐metabolic disorders including DM, DL, HTN, and obesity decreased significantly (*P* < 0.001 in all; *Table* 1).

**Table 1:** Clinical characteristics according to muscle strength quartiles

	Total	Q_1_	Q_2_	Q_3_	Q_4_	*P* for trend^a^
Unweighted *N*	13 502	3193	3354	3456	3499	
HGS (kg)^b^	31.8 ± 0.1	23.7 ± 0.2	29.7 ± 0.2	33.4 ± 0.2	37.5 ± 0.2	<0.001
HGS/BMI	1.35 ± 0.0	0.90 ± 0.0	1.19 ± 0.0	1.40 ± 0.0	1.71 ± 0.0	<0.001
Age (years)	45.6 ± 0.2	54.1 ± 0.5	48.2 ± 0.4	43.7 ± 0.3	39.7 ± 0.3	<0.001
BMI (kg/m^2^)	23.8 ± 0.0	26.2 ± 0.1	24.8 ± 0.1	23.6 ± 0.1	21.8 ± 0.1	<0.001
Waist circumference (cm)	82.0 ± 0.1	88.4 ± 0.2	84.4 ± 0.2	81.2 ± 0.2	76.7 ± 0.2	<0.001
Systolic BP (mmHg)	116.5 ± 0.2	121.9 ± 0.4	118.6 ± 0.3	115.4 ± 0.3	112.3 ± 0.3	<0.001
Diastolic BP (mmHg)	75.3 ± 0.1	75.5 ± 0.2	76.1 ± 0.2	75.6 ± 0.2	74.3 ± 0.2	<0.001
AST (IU/L)	22.0 ± 0.1	24.4 ± 0.3	23.0 ± 0.3	21.4 ± 0.2	20.2 ± 0.2	<0.001
ALT (IU/L)	22.0 ± 0.2	26.5 ± 0.6	24.2 ± 0.5	21.2 ± 0.3	18.2 ± 0.2	<0.001
Total cholesterol (mg/dL)	190.6 ± 0.4	192.1 ± 0.9	193.5 ± 0.8	191.3 ± 0.7	186.7 ± 0.7	<0.001
HDL (mg/dL)	51.0 ± 0.1	47.6 ± 0.2	49.7 ± 0.3	51.0 ± 0.2	54.2 ± 0.2	<0.001
TG (mg/dL)	138.2 ± 1.4	153.1 ± 2.6	150.5 ± 2.7	140.9 ± 2.6	117.0 ± 2.8	<0.001
LDL (mg/dL)	116.0 ± 0.5	117.9 ± 1.0	118.4 ± 1.1	116.5 ± 0.9	111.8 ± 1.0	<0.001
HbA1c (%)	5.6 ± 0.0	5.9 ± 0.0	5.7 ± 0.0	5.6 ± 0.0	5.5 ± 0.0	<0.001
HOMA‐IR	2.2 ± 0.0	3.2 ± 0.2	2.5 ± 0.1	2.0 ± 0.1	1.5 ± 0.0	<0.001
hs‐CRP (mg/L)	1.2 ± 0.0	1.7 ± 0.1	1.3 ± 0.1	1.1 ± 0.0	0.8 ± 0.0	<0.001
Diabetes mellitus (%)	10.7 ± 0.3	21.9 ± 0.9	12.8 ± 0.7	8.7 ± 0.6	3.6 ± 0.3	<0.001
Dyslipidaemia (%)	16.5 ± 0.4	23.0 ± 1.0	20.9 ± 0.8	15.8 ± 0.8	9.6 ± 0.6	<0.001
Hypertension (%)	24.1 ± 0.5	41.2 ± 1.2	29.6 ± 0.9	19.9 ± 0.9	12.4 ± 0.6	<0.001
Obesity (%)	33.6 ± 0.5	59.9 ± 1.1	44.8 ± 1.1	29.3 ± 0.9	11.6 ± 0.7	<0.001

ALT, alanine aminotransferase; AST, aspartate aminotransferase; BMI, body mass index; BP, blood pressure; HbA1c, glycated haemoglobin; HDL, high‐density lipoprotein; HGS, handgrip strength; HOMA‐IR, Homeostatic Model Assessment of Insulin Resistance; hs‐CRP, high‐sensitivity C‐reactive protein; LDL, low‐density lipoprotein; Q, quartile; Q_1_, the lowest quartile; Q_4_, the highest quartile; TG, triglycerides.

Muscle strength was calculated from mean HGS divided by BMI. Values are presented as mean or prevalence ± standard error.

^a^ From logistic and linear regression without any adjustment.

^b^ Handgrip strength of dominant arm.

### Association between non‐alcoholic fatty liver disease and low muscle strength

**Figure 2:**
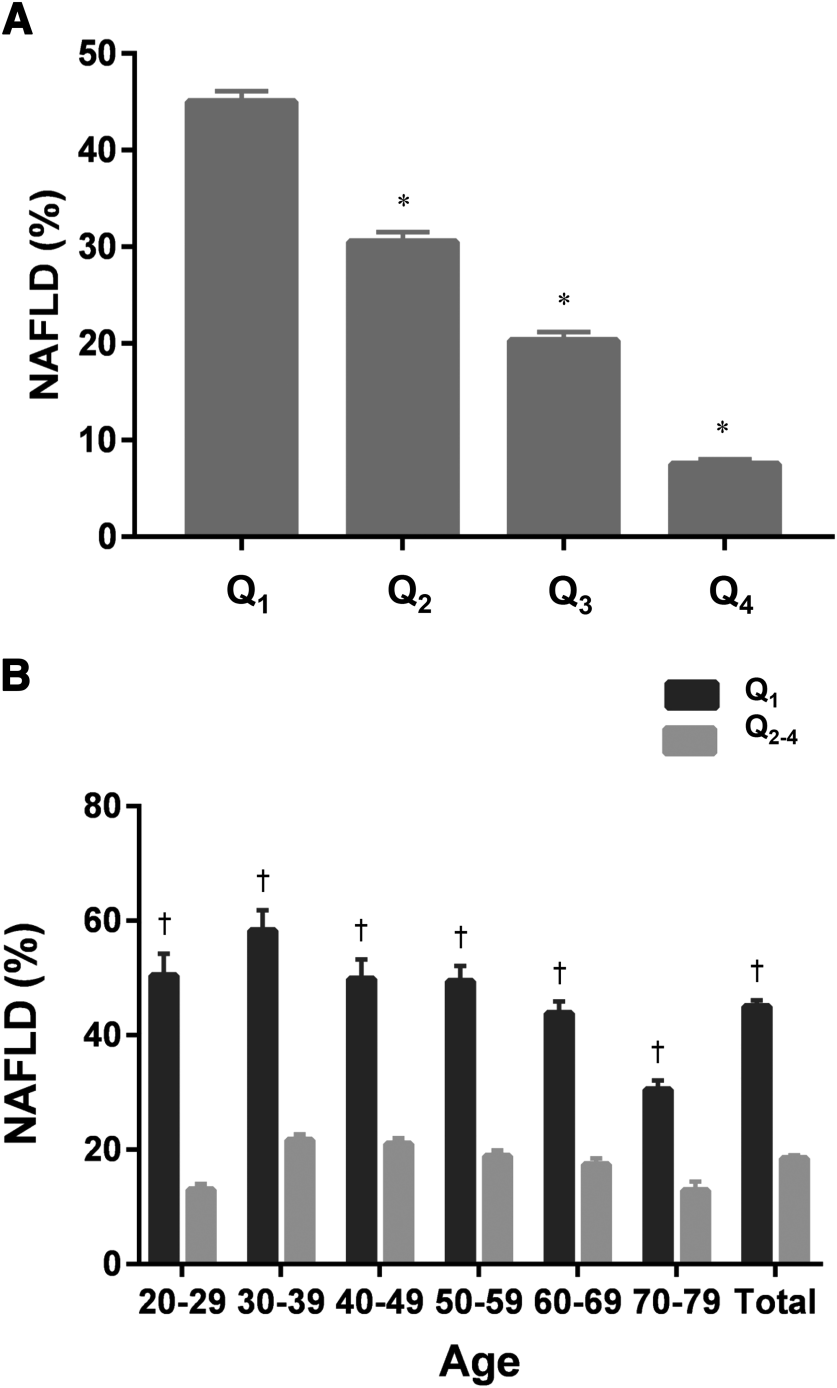
Prevalence of non‐alcoholic fatty liver disease (NAFLD) according to muscle strength. (A) The prevalence of NAFLD according to muscle strength quartiles in the entire study population. (B) The prevalence of NAFLD in 10 year age strata according to the presence of low muscle strength. ^*^Significantly lower compared with the Q_1_ (*P* < 0.05). ^†^Significantly higher compared with the rest of the groups (Q_2_, Q_3_, and Q_4_).

The prevalence of NAFLD was 45.0% (SE, 1.1%), 30.5% (SE, 1.0%), 20.3% (SE, 0.9%), and 7.5% (SE 0.5%) in the Q_1_, Q_2_, Q_3_, and Q_4_, respectively (*P* for trend <0.001; *Figure* 2A); LMS, defined as Q_1_, was associated with an increased prevalence of NAFLD (odds ratio [OR] 3.62, 95% confidence interval [CI] 3.25–4.03, *P* < 0.001). After adjustment for age, sex, obesity, DM, HTN, DL, and elevated hs‐CRP level, the association between LMS and NAFLD remained significant (OR 1.92, 95% CI 1.61–2.29, *P* < 0.001; Model 3 in *Table* 2). Even after additional adjustment for IR, LMS was associated with 1.66 times higher prevalence of NAFLD (OR 1.66, 95% CI 1.28–2.16, *P* < 0.001; Model 4 in *Table* 2).

**Table 2:** Association between NAFLD and low muscle strength

	NAFLD		
	OR	95% CI	*P*
Unadjusted	3.62	3.25–4.03	<0.001
Model 1	3.93	3.50–4.41	<0.001
Model 2	2.03	1.76–2.34	<0.001
Model 3	1.92	1.61–2.29	<0.001
Model 4	1.66	1.28–2.16	<0.001

CI, confidence interval; NAFLD, non‐alcoholic fatty liver disease; OR, odds ratio.

Low muscle strength was defined as the lowest quartile of muscle strength (handgrip strength/body mass index). Model 1, adjusted for age and sex; Model 2, adjusted for age, sex, and obesity; Model 3, with additional adjustment for the presence of diabetes mellitus, hypertension, dyslipidaemia, and elevated high‐sensitivity C‐reactive protein in addition to Model 2; Model 4, with additional adjustment for insulin resistance (Homeostatic Model Assessment of Insulin Resistance ≥2.5) in addition to Model 3.

Given a strong association between age and muscle strength, the prevalence of NAFLD according to muscle strength was investigated using a 10 year age stratification (Supporting Information, *Table* S1). We compared the prevalence of NAFLD in each quartile of muscle strength across each 10 year age strata in our sample group: 20–29, 30–39, 40–49, 50–59, 60–69, and 70–79 years. The prevalence of NAFLD in the LMS group (Q_1_) was significantly higher in all age strata groups than in the other groups (Q_2_, Q_3_, and Q_4_; *P* < 0.001 in all) (*Figure* 2B).

### The quantitative association between muscle strength and non‐alcoholic fatty liver disease

A dose–response relationship was observed between muscle strength and NAFLD (*Figure* 2A); the prevalence of NAFLD significantly decreased as the quartiles of muscle strength increased, compared with that in the Q_1_ (*Table* 3; *P* < 0.001 in all quartiles). For every 1 quartile (1Q) increment in muscle strength, the prevalence of NAFLD decreased by 51% (OR per 1Q increment 0.49, 95% CI 0.47–0.51, *P* < 0.001). The prevalence of NAFLD was significantly lower even in the Q_2_ group (i.e., the second lowest muscle strength group) compared with the Q_1_ group (OR for NAFLD 0.54, 95% CI 0.47–0.61, *P* < 0.001). After adjustment for age, sex, obesity, DM, HTN, DL, elevated hs‐CRP level, and IR, this quantitative association between muscle strength and the prevalence of NAFLD remained significant (Model 4 in *Table* 3). In Model 4, the prevalence of NAFLD decreased by 24% per 1Q increment in muscle strength (OR per 1Q increment 0.76, 95% CI 0.68–0.85, *P* < 0.001).

**Table 3:** Risk of NAFLD in each quartile of muscle strength

	NAFLD		
	OR	95% CI	*P*
Unadjusted			
Q_1_	(Reference)		
Q_2_	0.54	0.47–0.61	<0.001
Q_3_	0.31	0.27–0.36	<0.001
Q_4_	0.10	0.08–0.12	<0.001
Per 1Q	0.49	0.47–0.51	<0.001^*^
Model 1			
Q_1_	(Reference)		
Q_2_	0.49	0.43–0.56	<0.001
Q_3_	0.27	0.23–0.31	<0.001
Q_4_	0.08	0.07–0.10	<0.001
Per 1Q	0.45	0.43–0.48	<0.001^*^
Model 2			
Q_1_	(Reference)		
Q_2_	0.63	0.54–0.73	<0.001
Q_3_	0.49	0.41–0.59	<0.001
Q_4_	0.26	0.21–0.31	<0.001
Per 1Q	0.66	0.62–0.70	<0.001^*^
Model 3			
Q_1_	(Reference)		
Q_2_	0.60	0.49–0.74	<0.001
Q_3_	0.56	0.45–0.69	<0.001
Q_4_	0.30	0.23–0.39	<0.001
Per 1Q	0.70	0.65–0.76	<0.001^*^
Model 4			
Q_1_	(Reference)		
Q_2_	0.66	0.48–0.89	<0.001
Q_3_	0.67	0.49–0.93	0.005
Q_4_	0.38	0.27–0.54	0.003
Per 1Q	0.76	0.68–0.85	<0.001^*^

CI, confidence interval; NAFLD, non‐alcoholic fatty liver disease; OR, odds ratio.

Muscle strength was calculated from mean handgrip strength divided by body mass index. Q_1_, the lowest quartile; Q_4_, the highest quartile. Model 1, adjusted for age and sex; Model 2, adjusted for age, sex, and obesity; Model 3, with additional adjustment for the presence of diabetes mellitus, hypertension, dyslipidaemia, and elevated high‐sensitivity C‐reactive protein in addition to Model 2; Model 4, with additional adjustment for insulin resistance (Homeostatic Model Assessment of Insulin Resistance ≥2.5) in addition to Model 3.

^*^ *P* value for the test of trend of odds.

### Association between muscle strength and advanced fibrosis

**Figure 3:**
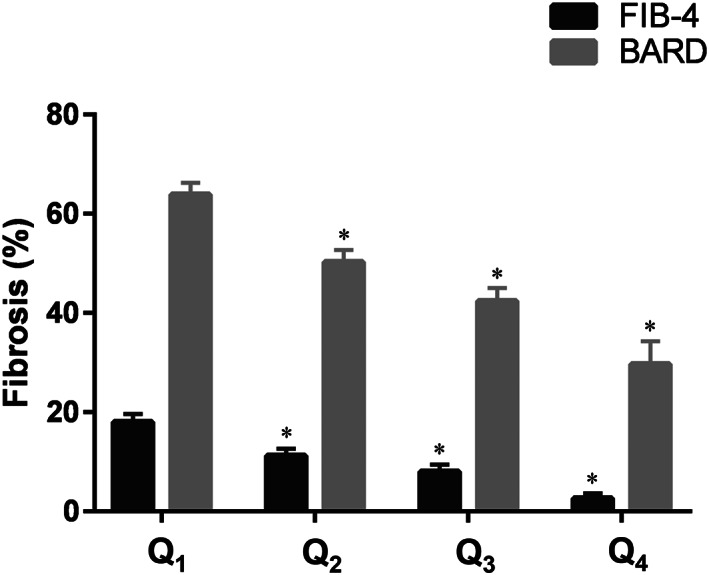
Prevalence of advanced fibrosis according to muscle strength. The prevalence of advanced fibrosis according to muscle strength quartiles. ^*^Significantly lower compared with the Q_1_ (*P* < 0.05). BARD, BARD score for non‐alcoholic fatty liver disease fibrosis; FIB‐4, fibrosis‐4 index; low muscle strength was defined as the lowest quartile (Q_1_) of muscle strength (handgrip strength/body mass index).

To investigate the association between muscle strength and advanced fibrosis, only individuals with NAFLD were selected (*n* = 2092). For these patients with NAFLD, both FIB‐4 and BARD scores were calculated to detect advanced fibrosis. The prevalence of advanced fibrosis in each quartile (Q_1_–Q_4_) showed a decreasing trend as muscle strength increased (*Figure* 3): Fibrosis_FIB4_, 18.0% (SE 1.6%), 11.2% (SE, 1.4%), 8.0% (SE, 1.4%), and 2.6% (SE, 1.0%) (*P* < 0.001); Fibrosis_BARD_, 63.9% (SE, 2.3%), 50.3% (SE, 2.4%), 42.4% (SE, 2.6%), and 29.7% (SE, 4.6%) (*P* < 0.001).

Low muscle strength was found to be significantly associated with an increased risk of advanced fibrosis (Fibrosis_FIB4_, OR 2.29, 95% CI 1.68–3.13, *P* < 0.001; Fibrosis_BARD_, OR 2.22, 95% CI 1.76–2.81, *P* < 0.001), which remained statistically significant after adjustment for age, sex, and obesity (Fibrosis_FIB4_, OR 1.50, 95% CI 1.06–2.12, *P* = 0.023; Fibrosis_BARD_, OR 1.57, 95% CI 1.21–2.04, *P* = 0.001; Model 2 in *Table* 4). Additional adjustment for DM, HTN, DL, and elevated hs‐CRP level did not attenuate the significant association between LMS and advanced fibrosis (Fibrosis_FIB4_, OR 1.66, 95% CI 1.01–2.49, *P* = 0.015; Fibrosis_BARD_, OR 1.81, 95% CI 1.30–2.51, *P* < 0.001; Model 3 in *Table* 4). However, adding IR to multivariable‐adjusted analysis weakened the association between LMS and advanced fibrosis; statistical significance was maintained for Fibrosis_BARD_ but not for Fibrosis_FIB4_ (Model 4 in *Table* 4).

**Table 4:** Risk of advanced fibrosis stratified by low muscle strength in subjects with non‐alcoholic fatty liver disease

	Fibrosis_FIB4_			Fibrosis_BARD_		
	OR	95% CI	*P*	OR	95% CI	*P*
Unadjusted	2.29	1.68–3.13	<0.001	2.22	1.76–2.81	<0.001
Model 1	1.56	1.11–2.20	0.011	1.90	1.48–2.44	<0.001
Model 2	1.50	1.06–2.12	0.023	1.57	1.21–2.04	0.001
Model 3	1.66	1.01–2.49	0.015	1.81	1.30–2.51	<0.001
Model 4	1.35	0.75–2.45	0.314	1.68	1.07–2.62	0.024

CI, confidence interval; Fibrosis_BARD_, BARD score ≥2; Fibrosis_FIB4_, fibrosis‐4 index >1.30; OR, odds ratio.

Low muscle strength was defined as the lowest quartile of muscle strength (handgrip strength/body mass index). Model 1, adjusted for age and sex; Model 2, adjusted for age, sex, and obesity; Model 3, with additional adjustment for the presence of diabetes mellitus, hypertension, dyslipidaemia, and elevated high‐sensitivity C‐reactive protein in addition to Model 2; Model 4, with additional adjustment for insulin resistance (Homeostatic Model Assessment of Insulin Resistance ≥2.5) in addition to Model 3.

In the current study, HOMA‐IR was available only in 671 subjects with NAFLD. In a stratified analysis according to IR, the association between LMS and Fibrosis_FIB4_ was more prominent in subjects with HOMA‐IR < 2.5 (unweighted *N* = 279; OR 3.05, 95% CI 1.27–7.30, *P* = 0.013) than in those with HOMA‐IR ≥ 2.5 (unweighted *N* = 392, OR 1.84, 95% CI 0.92–3.68, *P* = 0.085), and the same trend held true for Fibrosis_BARD_ (*Table* 5). Stratified analyses according to abdominal obesity, obesity, sex, or age did not reveal any differences in the association between LMS and advanced fibrosis (*Table* 5).

**Table 5:** Stratified association between advanced fibrosis and low muscle strength in subjects with non‐alcoholic fatty liver disease

	Fibrosis_FIB4_			Fibrosis_BARD_		
	OR	95% CI	*P*	OR	95% CI	*P*
HOMA‐IR						
<2.5	3.05	1.27–7.30	0.013	4.21	2.04–8.70	<0.001
≥2.5	1.84	0.92–3.68	0.085	1.71	1.09–2.67	0.019
Abdominal obesity						
No	1.95	0.98–3.87	0.057	2.17	1.31–3.60	0.003
Yes	2.42	1.72–3.41	<0.001	1.85	1.41–2.42	<0.001
Obesity						
No	2.42	0.86–6.81	0.092	2.40	1.01–5.43	0.036
Yes	2.20	1.58–3.06	<0.001	1.90	1.49–2.43	<0.001
Sex						
Male	2.43	1.51–3.90	<0.001	1.74	1.26–2.41	0.001
Female	2.05	1.35–3.10	0.001	2.73	1.88–3.97	<0.001
Age (years)						
<50	2.58	1.12–5.93	0.026	1.94	1.36–2.76	<0.001
≥50	1.60	1.12–2.28	0.010	2.11	1.52–2.94	<0.001

CI, confidence interval; Fibrosis_BARD_, BARD score ≥2; Fibrosis_FIB4_, fibrosis‐4 index >1.30; HOMA‐IR, Homeostatic Model Assessment of Insulin Resistance; OR, odds ratio.

Low muscle strength was defined as the lowest quartile of muscle strength (handgrip strength/body mass index). Each stratified analysis was performed using logistic regression without adjustment.

## Discussion

In the current study, muscle strength was inversely associated with the prevalence of NAFLD, irrespective of age. LMS was an independent risk factor for NAFLD regardless of age, sex, obesity, DM, HTN, DL, hs‐CRP level, and IR with the odds of NAFLD among individuals with LMS reaching 1.66. Moreover, there was a dose–response relationship between muscle strength and the prevalence of NAFLD; even individuals in the second lowest muscle strength group (Q_2_) had a significantly lower risk of NAFLD than those with LMS. Among those with NAFLD, LMS showed a significant association with advanced fibrosis independent of age, sex, and other metabolic derangements. However, additional adjustment for IR attenuated this association; LMS was not associated with Fibrosis_FIB4_ after adjustment for IR.

Sarcopenia is a syndrome characterized by loss of skeletal muscle mass and strength with an increased risk of adverse metabolic outcomes.^28^ Recently, LMS has become more widely recognized as a principal determinant of sarcopenia than low muscle mass, based on recent evidence that muscle strength may be more important to predict fracture, falling,^29^ cardiovascular disease, and all‐cause mortality.^30^ The most updated guideline from the European Working Group on Sarcopenia in Older People^31^ defined LMS as the key characteristic of sarcopenia. Diagnosis of sarcopenia can be made when LMS coexists with low muscle quantity, and the European Working Group on Sarcopenia in Older People recommends grip strength for a proxy measure of whole‐body strength. Grip strength measurement is also a quick and easy‐to‐obtain test and has been well validated as a tool to predict all causes of death.^30^

Several mechanisms may influence an inverse association between sarcopenia and NAFLD, including IR, inflammation, myokines, and decreased physical activity.^28^ In the current study, HOMA‐IR and hs‐CRP levels, as well as the prevalence of NAFLD, significantly increased as muscle strength decreased. Although obesity has been considered as the main pathophysiological factor leading to metabolic syndrome, including NAFLD, non‐obese individuals may also develop NAFLD.^32^ Skeletal muscle is the primary target organ of insulin‐mediated glucose disposal, and therefore, sarcopenia itself, independent of obesity, may aggravate IR.^33^ In the current study, the significant association between LMS and Fibrosis_FIB4_ lost its statistical significance after adjustment for IR, implicating that IR plays an important role in the relationship between LMS and advanced fibrosis in NAFLD. Interestingly, the stratified analysis showed that there was an association between LMS and advanced fibrosis in patients without IR compared with those with IR. Although IR plays a main pathogenic role in developing both NAFLD^34^, ^35^ and sarcopenia,^33^ the latter may increase the severity of NAFLD through IR‐independent mechanisms such as systemic inflammation.^36^, ^37^

Although several pharmacotherapeutic agents, such as vitamin E,^38^ thiazolidinedione,^39^ glucagon like peptide‐1 analogues,^40^ and farnesoid X receptor agonists,^41^ have shown positive results on the treatment of non‐alcoholic steatohepatitis, currently, there are no approved medications for NAFLD. The mainstay treatment is lifestyle intervention, focusing on weight loss. However, the achievement of the treatment goal (viz. a weight reduction of >10% of body weight^42^) to regress fibrosis is difficult to obtain for most patients with NAFLD. Considering the paucity in the effective treatment for NAFLD and the substantial NAFLD‐related morbidities, resistance training can be a promising treatment strategy against non‐alcoholic steatohepatitis and advanced fibrosis.

Resistance training has been shown to improve hepatic steatosis^43^, ^44^ and inflammation^45^ in patients with NAFLD, independently of weight loss. Resistance training may increase glycolysis and improve insulin sensitivity through hypertrophy of type II muscle fibres,^46^ increasing glucose transporter 4 expression,^47^ up‐regulation of AMP‐activated protein kinase and caveolins in type II muscle fibres,^48^ and increasing myokines that are beneficial to hepatic steatosis.^49^, ^50^ Irisin has been reported to have a protective effect on hepatic steatosis,^50^ and its level significantly increased after 8 weeks of initiating exercise in the resistance training group compared with that in the aerobic exercise group.^49^

There are several limitations in our study. First, data from liver imaging, such as ultrasonography and magnetic resonance imaging, as well as histological information, were not available in the current, population‐based study. Because only liver biopsy can distinguish steatohepatitis from simple steatosis, liver biopsy is considered a gold standard method but is hard to perform in primary centres due to its invasiveness and expensiveness. In addition, only a relatively small number of NAFLD subjects (671/2092, 32%) were subjected to HOMA‐IR tests, which might account for the lack of statistical significance in the association between LMS and advanced fibrosis adjusted for IR. Second, because of the cross‐sectional nature of the current study, causality cannot be determined between LMS and incident NAFLD or advanced fibrosis. Therefore, further prospective longitudinal cohort studies should be conducted to validate whether LMS is the real culprit of NAFLD and advanced fibrosis. Finally, because of insufficient muscle mass data in the current KNHANES analysis, we could not confirm whether the association between LMS and NAFLD persists independently of muscle mass. Although a previous study investigated the association between muscle mass and liver fibrosis using the KNHANES data between 2008 and 2011,^9^ it measured only muscle mass using dual‐energy X‐ray absorptiometry but not muscle strength. On the contrary, we used the KNHANES data between 2014 and 2016, which measured sarcopenia through the other way.

Collectively, we demonstrated the association between LMS and NAFLD or advanced fibrosis from the nationwide survey representing Korean population. LMS was significantly associated with an increased prevalence of NAFLD independently of age and BMI and was also closely associated with increased IR and higher prevalence of metabolic diseases. The association between LMS and advanced fibrosis in NAFLD subjects was found to be independent of metabolic diseases, such as DM, DL, and obesity, which seemed to be mediated through IR. Considering the cross‐sectional nature of the current study, further investigations using a large‐scale, histologically confirmed NAFLD cohort are warranted to draw a definite conclusion on the association between LMS and advanced fibrosis in NAFLD. Given that a substantial number of NAFLD subjects are non‐obese and there has been no approved pharmacotherapy, our study suggests the therapeutic role of resistance training, especially in lean subjects with NAFLD.

## Funding

This work was supported by a public healthcare research grant‐in‐aid from the Seoul Metropolitan Government Seoul National University Boramae Medical Center (04‐2019‐1), the National Research Foundation of Korea (NRF) grant funded by the Korean government [Ministry of Education, Science, and Technology (MEST)] (2016R1D1A1B04934590), and the Korea Health Technology R&D Project through the Korea Health Industry Development Institute (KHIDI) funded by the Ministry of Health and Welfare, Korea (HI17C0912).

## Author contributions

S.K. and B.K.K. designed the study and collected and analysed the data. S.K., M.K.M., W.K., and B.K.K. interpreted the data and wrote the manuscript. W.K. and B.K.K. critically reviewed and approved the final version of the manuscript.

## Conflict of interest

None declared.

## Supporting information


**Table S1.** Prevalence of nonalcoholic fatty liver disease according to muscle strength quartiles in 10‐year age strata
