# Supplementary material for: Association between muscle strength and advanced fibrosis in non‐alcoholic fatty liver disease: a Korean nationwide survey
Source: J Cachexia Sarcopenia Muscle. 2020 Jul 7;11(5):1232–41. doi: 10.1002/jcsm.12598 (PMC7567158; doi:10.1002/jcsm.12598)
Supplement: Supplementary file 1 — Table S1. Prevalence of nonalcoholic fatty liver disease according to muscle strength quartiles in 10‐year age strata [file JCSM-11-1232-s001.docx]

**Association between Muscle Strength and Advanced Fibrosis in Nonalcoholic Fatty Liver Disease: Korean Nationwide Survey**

Sunyoung Kang,**^1, 2^** Min Kyong Moon,**^1, 3^** Won Kim,^1,4^ Bo Kyung Koo**^1, 3^**

^1^Department of Internal Medicine, Seoul National University College of Medicine, Seoul, Korea

^2^Department of Internal Medicine, Seoul National University Hospital, Seoul, Korea

^3^Division of Endocrinology, Department of Internal Medicine, Seoul Metropolitan Government Boramae Medical Center, Seoul, Korea

^4^Division of Gastroenterology and Hepatology, Department of Internal Medicine, Seoul Metropolitan Government Boramae Medical Center, Seoul, Korea

**E-mail**

Sunyoung Kang: [sunyoungkang519@gmail.com](mailto:sunyoungkang519@gmail.com)

Min Kyoung Moon: [mkmoon@snu.ac.kr](mailto:mkmoon@snu.ac.kr)

**Corresponding Authors:**

Won Kim, M.D., Ph.D.

Division of Gastroenterology and Hepatology, Department of Internal Medicine, Seoul Metropolitan Government Seoul National University Boramae Medical Center

20, Boramae-ro 5-gil, Dongjak-gu, Seoul 07061, Republic of Korea

Phone: +82-2-870-2233; Fax: +82-2-831-2826; E-mail: [drwon1@snu.ac.kr](mailto:drwon1@snu.ac.kr)

Bo Kyung Koo, M.D., Ph.D.

Division of Endocrinology, Department of Internal Medicine, Seoul Metropolitan Government Seoul National University Boramae Medical Center

20 Boramae-ro 5-gil, Dongjak-Gu, Seoul 07061, Republic of Korea

Phone: +82-2-870-2225; Fax: +82-2-831-2826; E-mail: bokyungkoomd@gmail.com

**Table S1. Prevalence of nonalcoholic fatty liver disease according to muscle strength quartiles in 10-year age strata**

| Age (years) | Total | Q_1_ | Q_2_ | Q_3_ | Q_4_ | *P* for trend^1^ |
| --- | --- | --- | --- | --- | --- | --- |
| 20–29 | 18.1 ± 1.1% | 50.4 ± 3.8% | 27.6 ± 3.1% | 12.4 ± 1.7% | 6.0 ± 1.2% | <0.001 |
| 30–39 | 25.5 ± 1.0% | 58.3 ± 3.5% | 39.8 ± 2.6% | 26.8 ± 2.0% | 10.5 ± 1.1% | <0.001 |
| 40–49 | 24.4 ± 1.0% | 49.8 ± 3.4% | 34.7 ± 2.4% | 27.1 ± 1.8% | 8.4 ± 1.1% | <0.001 |
| 50–59 | 24.2 ± 1.0% | 49.4 ± 2.7% | 31.9 ± 2.0% | 18.6 ± 1.7% | 4.0 ± 0.9% | <0.001 |
| 60–69 | 25.9 ± 1.0% | 43.8 ± 2.1% | 24.5 ± 1.7% | 14.2 ± 1.7% | 5.2 ± 1.5% | <0.001 |
| 70–79 | 23.0 ± 1.1% | 30.5 ± 1.6% | 17.4 ± 2.2% | 6.6 ± 1.7% | - | <0.001 |
| Total | 23.5 ± 0.5% | 45.0 ± 1.1% | 30.5 ± 1.0% | 20.3 ± 0.9% | 7.50 ± 0.5% | <0.001 |

Muscle strength was calculated from mean hand grip strength divided by body mass index. Q_1_, the lowest quartile; Q_4_, the highest quartile

Values are presented as mean or prevalence ± standard error.

^1^From logistic regression without any adjustment
